# Rare comorbidity of colon cancer, hepatic malignant mesothelioma, and abdominal Ewing’s sarcoma: a case report

**DOI:** 10.3389/fonc.2025.1558347

**Published:** 2025-05-14

**Authors:** Yanru Li, Lukang Teng, Shupeng Wang, Xudong Wu, Tianchen Huang

**Affiliations:** ^1^ Henan University of Science and Technology School of Clinical Medicine, The First Affiliated Hospital of Henan University of Science and Technology, Luoyang, Henan, China; ^2^ Henan University of Science and Technology School of Clinical Medicine, The Four Affiliated Hospital of Henan University of Science and Technology, Anyang, Henan, China

**Keywords:** multiple primary carcinomas, hepatic malignant mesothelioma, abdominal Ewing’s sarcoma, comprehensive diagnosis and treatment, case report

## Abstract

We present a rare case of a patient with a history of colon cancer who subsequently developed hepatic mesothelioma and extraosseous Ewing’s sarcoma 10 years after surgery. The diagnosis of three completely different types of cancer in the same patient over a decade was confirmed through histopathological and immunohistochemical analysis. This case highlights the importance of clinicians maintaining a heightened awareness and clinical vigilance regarding multiple primary cancers, ensuring patients receive comprehensive diagnosis and treatment for improved prognosis and desired survival outcomes.

## 1 Introduction

Multiple primary carcinomas (MPC) refer to the simultaneous or consecutive occurrence of two or more primary malignant tumors in one or multiple organs of the same patient, with an incidence rate ranging from 0.73% to 11.70% (1). Among these, double cancer is more common, whereas triple and quadruple cancers are rare. It tends to occur in the digestive system, respiratory system, and urinary system, with the relationship between the second primary cancer lesion and the first primary cancer lesion being close. Here, we report a case of triple cancer involving colon cancer, hepatic malignant mesothelioma, and extracavitary Ewing’s sarcoma, and conduct a retrospective analysis in conjunction with existing literature to guide clinical treatment.

## 2 Case report

### 2.1 Patient introduction

The patient was a 42-year-old male who visited the First Affiliated Hospital of Henan University of Science and Technology on August 3, 2024, due to intermittent right upper abdominal pain for one week. He reported no other symptoms.

### 2.2 Physical examination

He had mild tenderness in the right upper abdomen, with no other abnormalities.

### 2.3 Past medical history

He has a history of hepatitis B spanning two years and has been treated with oral entecavir. Six years ago, the patient underwent a “radical resection of sigmoid colon cancer.” Following surgery, the patient received adjuvant chemotherapy comprising oxaliplatin and capecitabine (I will omit the dosage and frequency).

### 2.4 Personal and family history

The patient has a 5-year history of smoking, no history of asbestos exposure, and no family history of colorectal cancer.

### 2.5 Laboratory tests

The laboratory examination results are as follows: Red blood cell count of 1.15x10^12/L, hemoglobin count of 31g/L, platelet count of 345x10^9/L. Carcinoembryonic antigen level of 1.71ng/ml, cancer antigen 125 level of 688U/ml, alpha-fetoprotein level of 2.00ng/ml.

### 2.6 Imaging studies

**Figure 1:**
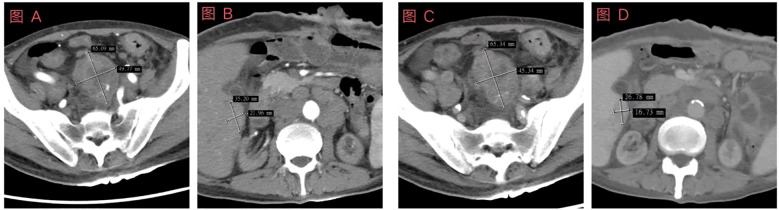
Schematic diagram of enhanced computed tomography (CT) examination results prior to the first chemotherapy. **(A)** Enhanced abdominal CT image showing arterial pelvic lesions; **(B)** Contrast-enhanced abdominal CT image of the liver during the arterial phase; **(C)** Enhanced abdominal CT image depicting venous pelvic lesions; **(D)** Contrast-enhanced abdominal CT image of the liver during the portal venous phase. In the abdominal and pelvic cavities, some lesions are fused and exhibit uneven enhancement.

Upon admission, the enhanced CT scans revealed: A small cyst in the right lobe of the liver was poorly visualized during the arterial phase. Patchy low-density lesions were noted in the right lobe during the venous phase, with indistinct borders. Some intestinal loops within the abdominal cavity exhibited postoperative changes, and the wall of the sigmoid colon-rectum was slightly thickened, presenting a mass-like appearance with heterogeneous enhancement. Furthermore, multiple enlarged lymph nodes were present in the hepatoduodenal ligament and abdominopelvic cavity, with some being fused and showing heterogeneous enhancement (**Figure 1**).

### 2.7 Pathological examination

**Figure 2:**
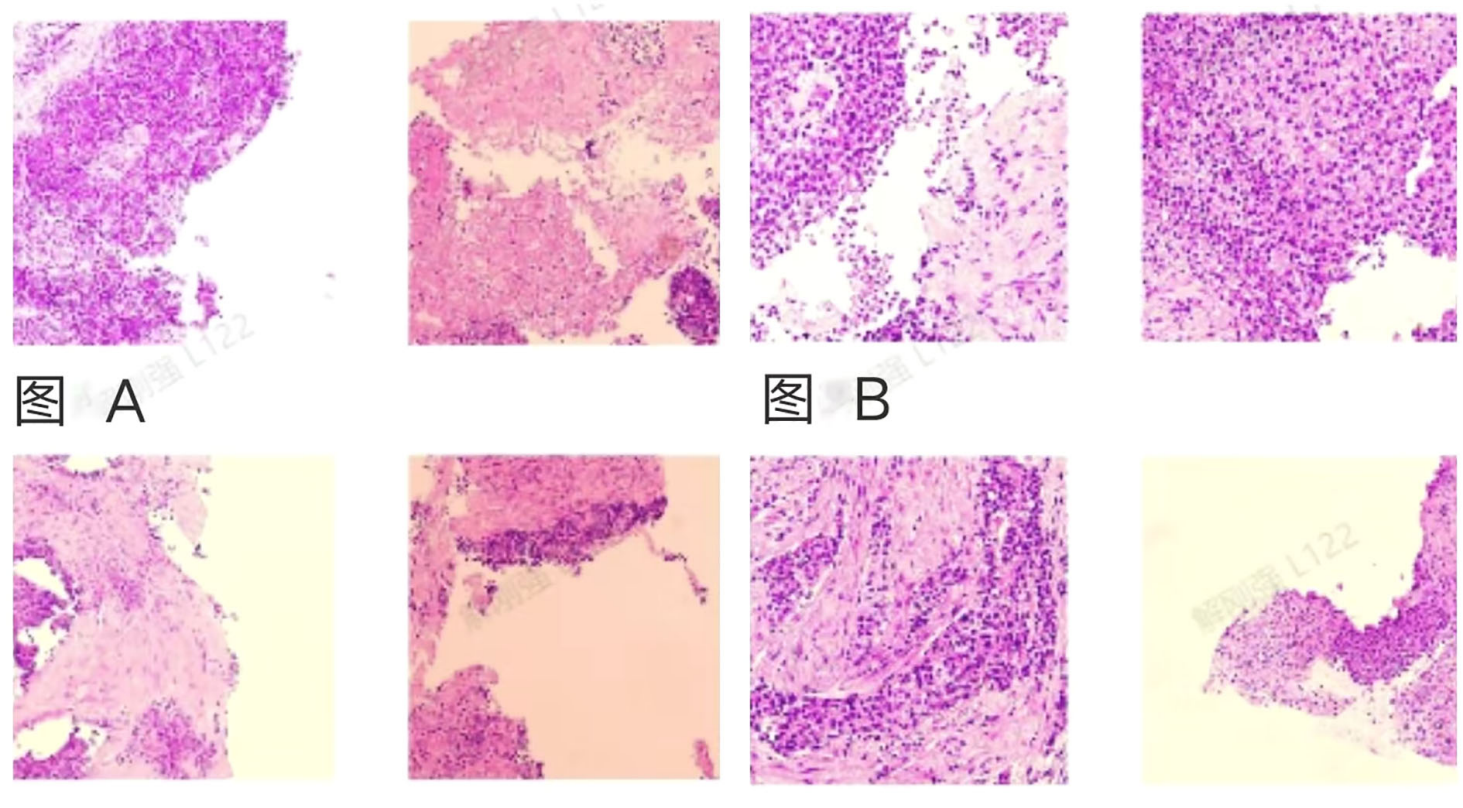
Schematic diagram of pathological results for pelvic mass and liver mass. **(A)** Optical microscope image of the pelvic mass, consistent with extraosseous Ewing sarcoma. **(B)** Optical microscope image of the liver mass, which tends to originate from malignant mesothelioma. Considering the medical history and immunohistochemical results, this does not support a colonic origin.

Ultrasound-guided biopsy of a pelvic mass and liver mass, postoperative pathological report of the pelvic mass: consistent with extraosseous Ewing’s sarcoma. Immunohistochemical results: Ki-67 (approximately 80%+), Vim(+), CD99(+), INI-1(+). Liver mass pathological report: Tends to originate from malignant mesothelioma. Immunohistochemical results: WT-1(+), CR(+), CyclinD1(+), Ki-67(30%+), FLI-1(+), CK5/6(+), D2-40(-) (**Figure 2**).

### 2.8 Treatment plan and follow-up

**Figure 3:**
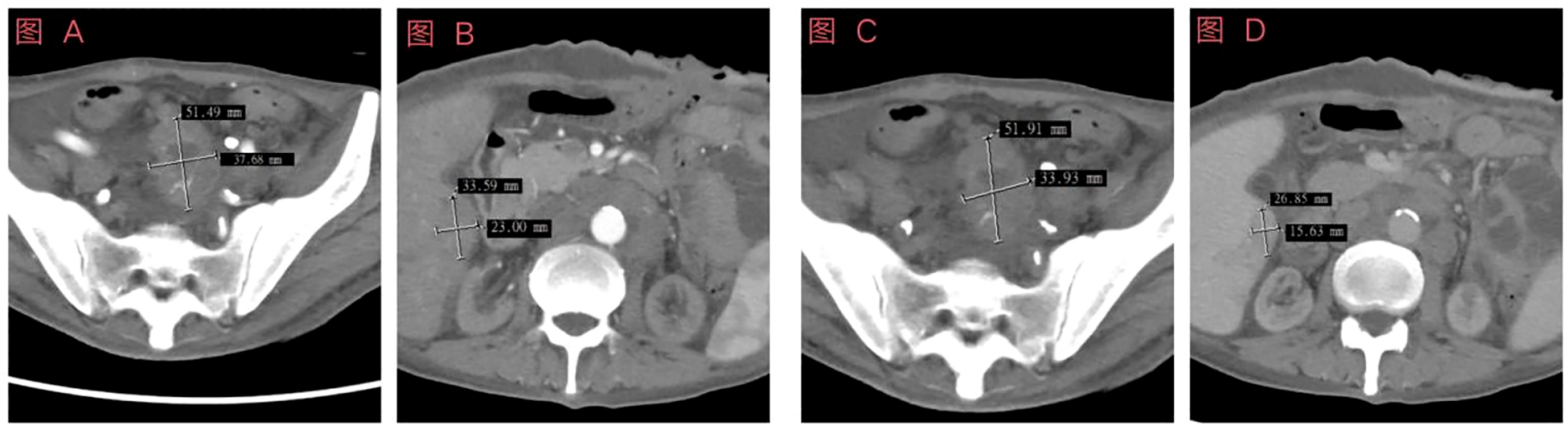
Schematic diagram of enhanced computed tomography (CT) examination results prior to the fourth chemotherapy session. **(A)** Enhanced abdominal CT image showing arterial pelvic lesions; **(B)** Contrast-enhanced abdominal CT image of the liver during the arterial phase; **(C)** Enhanced abdominal CT image depicting venous pelvic lesions; **(D)** Contrast-enhanced abdominal CT image of the liver during the portal venous phase.

**Figure 4:**
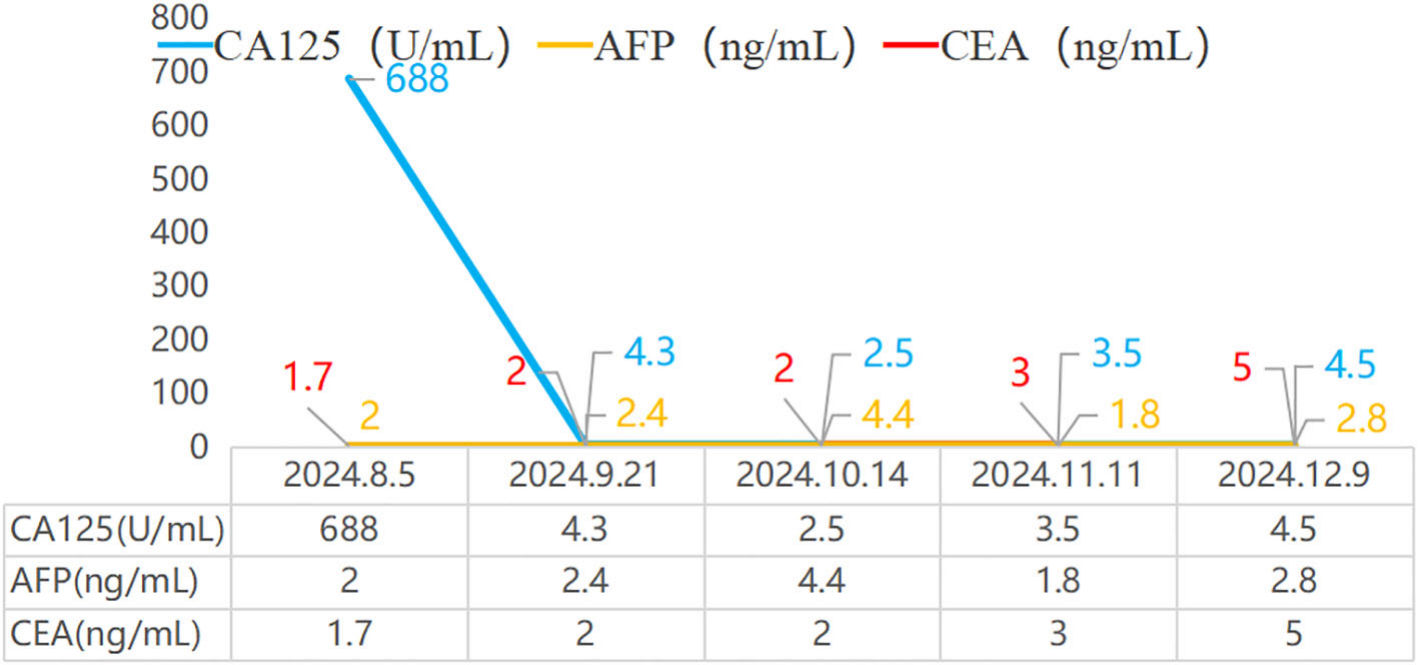
Trend chart of cancer antigen 125, alpha-fetoprotein, and carcinoembryonic antigen indicators during disease follow-up. CA125: Cancer Antigen 125; AFP: Alpha-fetoprotein; CEA: Carcinoembryonic Antigen.

After considering the patient’s medical history and immunohistochemical results, colon cancer is temporarily ruled out. Based on the latest treatment guidelines, the following treatment plan has been formulated: Vincristine (1.5mg/m^2^,IV, D1, D8, D15) + cyclophosphamide (1.2g/m^2^, IV over 1 hour, D1) + doxorubicin (30mg/m^2^, IV over 6 hours, D1~D2); pemetrexed (500mg/m^2^, IV, D1) + cisplatin (75mg/m^2^, IV, D1), a total of 4 cycles of treatment, Upon re-examination of the abdominal CT, it was found that the pelvic mass had decreased in size, and there was no significant change in the mesothelioma of the liver (**Figure 3**).The treatment plan for liver mesothelioma was adjusted to pemetrexed (800mg/m^2^, IV, D1) + cisplatin (40mg/m^2^, IV, D1~D2) + bevacizumab (400mg/m^2^, IV, D1), the patient is currently on the 1st cycle of medication, the condition is stable, and further follow-up is ongoing. In addition, We provided key indicators during the follow-up period in **Figure 4** and **Table 1**.

**Table 1:** Trend chart of cancer antigen 125, alpha-fetoprotein, and carcinoembryonic antigen indicators during disease follow-up. CA125: Cancer Antigen125; AFP: Alpha-fetoprotein; CEA: Carcinoembryonic Antigen.

	2024.8.5	2024.9.21	2024.10.14	2024.11.11	2024.12.9
CA125(U/mL)	688	4.3	2.5	3.5	4.5
AFP(ng/mL)	2	2.4	4.4	1.8	2.8
CEA(ng/mL)	1.7	2	2	3	5

## 3 Discussion

MPC is classified based on the interval between the onset of diseases into synchronous (from the onset of the first primary cancer < 6 months) and metachronous (≥ 6 months) (2).

Malignant mesothelioma (MM) is a type of occupational tumor associated with asbestos exposure, commonly occurring in the peritoneum, pleura, and pericardium. It has a high mortality rate and a long latency period of 20 to 40 years, with a median survival time of only 12 to 18 months (3). Extraosseous Ewing’s Sarcoma (EES) is a form of Ewing’s sarcoma that develops outside of bone tissue. It exhibits a high degree of malignancy, and early diagnosis is challenging, primarily depending on histopathological and immunohistochemical analyses. Treatment commonly involves a combination of surgery, radiation therapy, and chemotherapy. In this case, following a comprehensive diagnostic evaluation that included blood tests, tumor marker measurements, computed tomography imaging, and pathological biopsy, the patient was ultimately diagnosed with colon cancer, malignant peritoneal mesothelioma, and abdominal Ewing’s sarcoma. Both the pathological biopsy and CT scan results ruled out the possibility of the tumors originating from the same primary cancer. Following the latest clinical guidelines, the patient received adjuvant chemotherapy for colon cancer and first-line treatment for Ewing’s sarcoma and malignant mesothelioma. The treatment plan was adjusted in a timely manner based on the results of regular follow-up examinations.

Clinically, both Japan and China are facing the issue of high incidence of gastrointestinal tumors, among which multiple primary cancers (MPC) related to gastrointestinal tumors have the highest incidence rate among all types of MPC (4). There have been quite abundant research reports on multiple primary cancers (MPC), especially abdominal tumors, which usually occur in organs such as the liver, gastrointestinal tract, pancreas, and ovaries. Nonetheless, it remains relatively rare for patients to successively suffer from three different types of cancer, such as colon cancer, liver malignant mesothelioma, and abdominal Ewing’s sarcoma. According to existing research, the etiology of MPC is complex and diverse, but its exact mechanism has not been fully elucidated. It is generally believed that the pathogenic factors of the second and third primary tumors are similar to those of the first primary tumor.

Based on the current literature, the pathogenic factors of MPC mainly include: First, genetic and hereditary factors: Many genetic variations are closely linked to the mechanisms of cancer production, and these variations may stem from changes in different genes. Secondly, immune dysfunction: Patients diagnosed with malignant tumors often experience anxiety, depression, and other negative emotions. Long-term psychological stress and the use of immunosuppressants lead to significant changes in immune responses (5), damaging the immune system and reducing its surveillance ability against cancer cells, thus significantly increasing the risk of developing multiple cancers. Thirdly, unhealthy lifestyles: Studies indicate that smoking and alcohol-related primary and secondary malignant tumors account for up to 35% among survivors of malignant tumors in the United States (6). Fourth, Iatrogenic factors: Long-term use of certain chemotherapeutic drugs can cause gene mutations, thereby inducing multiple cancers.

In this case, the patient successively experienced three different types of gastrointestinal malignancies. Through thorough collection of medical history, we learned that the patient had neither a family history of cancer nor exposure to harmful substances such as asbestos or dust. After surgery, the patient underwent genetic testing, but no mutated genes were found, and the microsatellite status remained stable. A study involving 2,025 Chinese patients with multiple primary colorectal cancers revealed that the proportion of patients with a family history of malignant tumors was 13.1% (7). Furthermore, the study indicated that a significant proportion of patients with multiple primary cancers carry hereditary pathogenic variants, such as BRCA1, BRCA2, K-RAS (8). Hereditary pathogenic variants refer to gene mutations present in germ cells, which can cause diseases and be inherited by offspring, thereby increasing the risk of cancer in future generations.

Studies indicate that MPC display significant anatomical clustering, with high incidence areas primarily focused in two domains: firstly, the site of the initial primary tumor and its corresponding paired organs (such as the lungs, kidneys, and other bilateral organs); secondly, related organs within the same system as the primary tumor. MPC originating from the colorectum is relatively rare in clinical settings. A retrospective analysis of 758 colorectal cancer patients revealed that 33 of these patients developed extracolonic MPC, comprising 21 cases of metachronous MPC and 12 cases of synchronous MPC. In the majority of cases, the second primary tumor was the principal finding, with only 2 patients having more than 3 tumors. Among the second primary tumors, gastric cancer constituted 36.4%, thyroid cancer and prostate cancer each accounted for 15.1%, and esophageal cancer made up 6.0% (9). It is important to note that prolonged exposure to carcinogenic factors such as smoking and drinking, particularly in the digestive and respiratory tracts, significantly increases the risk of MPC. The patient in this case had a 5-year history of smoking, and this persistent risk factor may be associated with the occurrence of his MPC.

After a decade-long single-center retrospective study, scholars revealed that the incidence of MPC exhibits a significant age-related pattern. The findings indicate that the incidence rate of MPC increases with age, peaking between 50 to 60 years old, with the proportion of MPC reaching as high as 33.3% in patients aged 60 and above. The average age of the patients in this study was 59 years old. Further analysis points out that among patients first diagnosed with a tumor between the ages of 60 to 69, the risk of developing a second primary tumor within ten years can increase to 13% (10). Another research team conducted a large-scale population cohort study that also revealed gender differences. This epidemiological survey showed that the relative risk of MPC in males is significantly higher than in females (11).

Modern radiation therapy techniques have undergone revolutionary advancements, including precise targeted treatments and conformal optimization. Nonetheless, the potential carcinogenic effects still merit our vigilance. Secondary radiation products generated by radiation therapy can significantly increase the risk of MPC (12). In the field of chemotherapy, certain cytotoxic drugs—including alkylating agents, topoisomerase II inhibitors, and antimetabolites—can induce malignant transformation through various mechanisms: 1) the formation of DNA-protein crosslink complexes; 2) the initiation of double-strand DNA breaks; 3) the activation of proto-oncogene signaling pathways; 4) the induction of chromosomal unbalanced translocations. Clinical data suggests that long-term treatment with cytotoxic drugs in cancer patients may lead to the development of second primary tumors (13, 14).

In clinical practice, some clinicians still adhere to a habitual way of thinking, believing that patients cannot have multiple primary cancers simultaneously. This often leads to the possibility of multiple primary cancers (MPC) being overlooked, resulting in missed diagnoses. During the diagnostic process, clinicians frequently default to considering the recurrence or metastasis of tumors rather than the potential coexistence of multiple MPCs. In this case, the patient was diagnosed with three primary malignant tumors in different locations over a period of ten years, and these cancers occurred in different organs, which is a rare occurrence for the same individual. However, the relationship between these factors remains unclear and necessitates further study.

Relevant studies have revealed that the prognosis of patients with MPC is influenced by a complex interplay of factors, including the clinical pathological stage of the tumor, the level of differentiation, the characteristics of the site distribution, the time interval between the occurrence of two primary tumors, the treatment modalities received, and the age of the patient (15). Early and precise diagnosis, as well as standardized treatment including radical surgery, adjuvant chemotherapy, and radiotherapy, are all closely linked to the patient’s prognosis. It is evident that early detection, precise diagnosis, and timely treatment play a decisive role in improving the prognosis of patients with MPC. To effectively prevent and screen for MPC, medical personnel must possess solid professional knowledge. By strengthening medical staff’s understanding of the importance of MPC risk assessment and screening for high-risk populations, the accessibility of early diagnosis and treatment for patients can be improved. For patients who have achieved long-term survival from their first primary tumor, doctors should comprehensively understand their family medical history, work environment conditions, characteristics of lifestyle habits, and previous exposure to radiotherapy and chemotherapy and endocrine therapy, to accurately assess their risk of developing a second primary tumor. Therefore, enhancing clinicians’ awareness of the importance of conducting risk assessments for multiple primary cancers and screening high-risk populations is key to ensuring that MPC patients receive early diagnosis and treatment.

## Data Availability

The original contributions presented in the study are included in the article/supplementary material. Further inquiries can be directed to the corresponding author.
